# Association between C-reactive protein-triglyceride glucose index and abnormal BMD in middle-aged and elderly patients with type 2 diabetes mellitus: a cross-sectional study

**DOI:** 10.3389/fmed.2025.1615596

**Published:** 2025-10-16

**Authors:** Yuan Zhang, Yali Jing

**Affiliations:** ^1^Department of Endocrinology, Endocrine and Metabolic Disease Medical Center, Nanjing Drum Tower Hospital Clinical College of Nanjing University of Chinese Medicine, Branch of National Clinical Research Centre for Metabolic Diseases, Nanjing, China; ^2^Department of Endocrinology, Endocrine and Metabolic Disease Medical Center, Nanjing Drum Tower Hospital, Affiliated Hospital of Medical School, Nanjing University, Branch of National Clinical Research Centre for Metabolic Diseases, Nanjing, China

**Keywords:** osteopenia, osteoporosis, CTI, type 2 diabetes mellitus, aging

## Abstract

**Background and aims:**

Bone loss is an irreversible physiological change occurring with age. Type 2 diabetes mellitus (T2DM) is associated with poor bone health, and insulin resistance (IR) and inflammation are common pathologic mechanisms in T2DM and osteopenia/osteoporosis (OP). C-reactive protein-triglyceride glucose index (CTI), a novel marker of IR and inflammation, has been Investigated in various diseases. However, its potential association with the incidence of T2DM combined osteopenia/OP in T2DM remains unclear. This study aimed to investigate the association between the CTI and osteopenia/OP in middle-aged and elderly patients with T2DM.

**Methods:**

This retrospective cross-sectional study analyzed 847 middle-aged and elderly patients with T2DM. The CTI was calculated as follows: 0.412 × Ln [CRP (mg/L)]+Ln [TG (mg/dl) × FPG (mg/dl))/2. Spearman correlation analysis was employed to explore the connection among CTI with bone metabolic parameters in T2DM. Further, CTI was included in the logistic regression model as a continuous and categorical variable, respectively, to assess the association between this index and osteopenia/OP in T2DM. Additionally, the operating Characteristic (ROC) curve analysis was adopted to examine the predictive efficacy of the CTI for osteopenia/OP in T2DM.

**Results:**

This study found that the CTI level was significant higher in osteopenia/OP patients than normal subjects with T2DM (9.179 ± 0.500 vs. 9.684 ± 0.514, *p* < 0.001). Participants stratified by CTI quartiles showed a progressively increased prevalence of osteopenia/OP with higher CTI levels (22.6%−77.7%, *p* for trend < 0.001). In Spearman correlation analysis, a remarkably negative correlation was observed between CTI and bone mineral density (BMD) measures in middle-aged and elderly patients with T2DM. When analyzed CTI as continuous variable, after adjustment for the impact of various covariates, there was a significant relationship between CTI and the risk of osteopenia/OP (OR 7.277, 95% CI: 4.602–11.507; *p* < 0.001). When categorized CTI into quartiles, it still showed a statistically significant association with osteopenia/OP. The area under the ROC curve (AUC) showed a modest standalone predictive ability of 0.758 (95% CI: 0.7254–0.7897; specificity = 69.3%; sensitivity = 71.2%).

**Conclusion:**

A high CTI is associated with an increased risk of osteopenia/OP in middle-aged and elderly patients with T2DM. Incorporation of routine CTI monitoring into clinical practice may facilitate early detection of high-risk individuals, promote timely intervention, and reduce the burden of T2DM combined with bone metabolic disease.

## Introduction

Osteoporosis (OP) is a metabolic bone disease characterized by decreased bone mineral density (BMD) and destruction of bone microstructure, leading to increased bone fragility and susceptibility to fracture (1). It was estimated that the global prevalence of osteoporosis and osteopenia is 19.7 and 40.4%, respectively. Among adults aged over 50 years, the prevalence of OP ranges from one- to two-thirds, with postmenopausal women being at particularly high risk (2–5). The pathogenesis of osteoporosis is complex and is the result of a combination of factors. The core pathophysiologic mechanism is the imbalance between bone resorption and bone formation, which leads to a significant increase in bone fragility and thus a high risk of fracture. While osteopenia is the early stage of OP, fracture is the most serious clinical consequence, with extremely negative effects on the quality of life and life expectancy of patients (6). In addition, OP shares common pathogenic mechanisms, such as inflammatory response and oxidative stress, with a variety of chronic diseases, including diabetes mellitus (DM) and cardiovascular diseases (CVDs), which interact with each other and further aggravate the health burden of patients (7).

It is estimated that the global prevalence of diabetes will rise to 12.2% (783.2 million people) by 2045, with a parallel increase in the prevalence of type 2 diabetes mellitus (T2DM) in middle-aged and older population (8). Studies have shown that clinical characteristics of T2DM, including longer duration of T2DM, exogenous insulin therapy, vascular complications, and poorer glycemic control, are associated with lower bone turnover and increased fractures, particularly in the hip and distal limbs, either directly or due to negative effects on the skeleton (9, 10).

Insulin resistance (IR), physiologically defined as a state of reduced responsiveness of insulin-targeted tissues (e.g., skeletal muscle, liver, adipose tissue) to high physiologic insulin levels, is considered to be a causative driver of many modern diseases, including T2DM, metabolic syndrome, and atherosclerosis (11, 12). Emerging evidence suggested a significant interaction between OP and IR. OP may contribute to the development of IR, which in turn increases the risk of metabolic diseases and death. Similarly, IR may also play a key role in the pathogenesis of bone metabolic imbalances, primarily by disrupting the balance of osteoblast-mediated bone formation and osteoclast-mediated bone resorption, as well as altering the secretion of bone turnover markers (10, 11, 13, 14). In recent years, there has been a growing focus on the role of novel surrogate indicators of insulin sensitivity in clinical practice, such as the triglyceride–glucose (TyG) index, combining triglyceride (TG) and fasting plasma glucose (FPG), has shown promise as a predictive tool (15, 16). In addition, inflammation is strongly associated with poor prognosis in metabolic and cardiovascular diseases (17). Chronic inflammatory states can lead to micro/macrovascular complications of diabetes, and inflammatory factors can act directly or indirectly on osteoblasts, leading to bone loss and increased fracture risk. C-reactive protein (CRP), as a non-specific marker of inflammation, has emerged as a promising biomarker for assessing the level of inflammation (18).

C-reactive protein-triglyceride glucose index (CTI) is a novel marker that comprehensively assesses the severity of IR and inflammation, providing an innovative perspective on the evaluation of certain diseases risk (19, 20). Both T2DM and osteoporosis/osteopenia are multifactorial diseases, therefore, exploring the association between CTI and the risk of osteopenia/OP in middle-aged and elderly patients with T2DM, and then taking effective preventive measures, is crucial for disease prevention. We hypothesized that higher CTI values are associated with a higher risk of reduced BMD, and the findings of this study are anticipated to bridge the gap between low BMD and markers of IR-inflammation function and have clinical implications for the management of osteopenia/OP in middle-aged and elderly T2DM patients, thereby improving screening or early detection of osteopenia/OP in this population.

## Materials and methods

### Study design and population

This retrospective cross-sectional study included 847 T2DM patients aged 51–80 years who were hospitalized in the Department of Endocrinology of Nanjing Drum Tower Hospital from 2023 to 2024. This study followed the Declaration of Helsinki thoroughly and received approval from the institutional review board (Approval No. 2024-603-03), and informed consent was not required due to its retrospective nature. T2DM was diagnosed according to the 2003 American Diabetes Association criteria (21). Inclusion criteria were as follows: (1) postmenopausal women with T2DM or men with T2DM over 50 years old; (2) patients with complete data. Exclusion criteria are as follows: (1) presence of type 1 diabetes, other types of diabetes; (2) acute complications of diabetes, such as diabetic hyperosmotic hyperglycemia syndrome, diabetic ketoacidosis, and hypoglycemia; (3) patients with diseases which seriously affect bone metabolism and lead to secondary osteoporosis, such as past or present autoimmune diseases (rheumatoid arthritis), hematological diseases, hyper-/hypo-thyroidism, primary hyper-/hypo-parathyroidism, rickets, cancer, and Cushing syndrome; (4) medical treatment that could affect bone metabolism, including thiazolidinediones, calcium, calcitriol, osteoporosis medication (anabolic or antiresorptive) and glucocorticoid; and (5) disabled patients who cannot move or bedridden for a long time.

### Clinical measurements and calculations

General data were collected for all enrolled participants from the hospital's electronic medical records, including age, gender, diabetes duration, systolic blood pressure (SBP), diastolic blood pressure (DBP) and treatment with anti-diabetic drug, including insulin and its analogs, biguanides, glucagon-like peptide-1 (GLP-1) receptor agonists, Dipeptidyl peptidase-4 (DPP-IV) inhibitors, sodium-glucose co-transporter-2 (SGLT-2) inhibitors, insulin secretagogues, and α-Glucosidase inhibitors. Participants underwent height and weight measurements, with body mass index (BMI) subsequently calculated. All blood samples were collected in the early morning after an 8-h fasting period and subsequently transferred to the clinical laboratory of our hospital. The following biochemical variables were determined: glycated hemoglobin (HbA1c), FPG, 2-h post-load plasma glucose (2h PG), fasting insulin (FINS), fasting C-peptide (FCP), alanine aminotransferase (ALT), aspartate aminotransferase (AST), γ-glutamyl transferase (γ-GT), alkaline phosphatase (AKP), blood urea nitrogen (BUN), creatinine (Cr), uric acid (UA), triglyceride (TC), TG, high-density lipoprotein cholesterol (HDL-C), low-density lipoprotein cholesterol (LDL-C), serum calcium (Ca), serum phosphorus (P), estimated glomerular filtration rate (eGFR), CRP, hemoglobin (HGB), osteocalcin (OC), Beta-isomer of the carboxy-terminal cross-linked telopeptide of type I collagen (CTX), procollagen type I N-terminal propeptide (PINP), and 25-Hydroxyvitamin D (25 (OH) D), insulin-like growth factor-1 (IGF-1) and parathyroid hormone (PTH). Meanwhile, we calculated the CTI = 0.412 × Ln [CRP (mg/L)) + Ln [TG (mg/dl) × FPG (mg/dl)/2] (22).

Standard modules were used for quality control before daily operation, and the practitioner was blinded to the clinical information. Bioelectrical impedance analysis (BIA, InBody Co., Ltd) method was adopted to determine the body fat mass (BFM), soft lean mass (SLM), fat-free mass (FFM), waist-to-hip ratio (WHR), visceral fat area (VFA), appendicular skeletal muscle mass (ASM) of patients and to calculate the fat-free mass index (FFMI) and subsequent appendicular skeletal muscle index (SMI, kg/m^2^). Bone mineral density (g/cm^2^) was measured using dual energy X-ray absorptiometry (Lunar iDXA, USA). Measurements were made at three sites in each patient: femoral neck (FN), left hip (LH) and lumbar spine (LS). The diagnosis of osteoporosis was also based on WHO criteria, T-score ≤ -2.5 for osteoporosis, between −2.5 and −1.0 for osteopenia, T-score >-1.0 for normality and T-score ≤ -1.0 for abnormal bone BMD (23).

### Statistical analysis

All data were examined by adopting the program SPSS Statistics software version 27.0 (IBM, Corporation, Armonk, NY, USA). First, according to T-score, subjects were categorized into normal group (T-score ≥-1.0) and abnormal BMD group (T-score < -1.0), including osteopenia/osteoporosis, to analyze the basic characteristics; then, the CTI levels were segmented into quartiles, and compared the trends of the various indicators in Q1, Q2, Q3 and Q4 groups. The continuous variables in accordance with normal distribution were expressed by (mean ± standard deviation), and the comparison between groups was expressed by independent sample *t*-test and ANOVA test, while continuous variables with a non-normal distribution were expressed by medians [interquartile ranges (IQR)], and the comparison between groups was expressed by the Mann–Whitney *U* test and Kruskal–Wallis test. The categorical variables were reported in the form of percentages and comparative analyses of differences were performed using the Chi-square test or Fisher's exact test. Spearman correlation analysis was used to examine the correlation between CTI and bone metabolism and BMD parameters. The logistic regression was constructed to investigate the correlation between CTI with the risk of osteopenia/OP. In addition, the capacity of the CTI to forecast T-score < -1.0 for abnormal bone metabolism was analyzed using receiver operating characteristic (ROC) curves, and the results are shown as an area under the curve (AUC). The results were evaluated within a 95% confidence interval (CI) and at a significance level of two-sided *p*-value < 0.05. Statistics were considered significant when *p* < 0.05.

## Results

**Figure 1:**
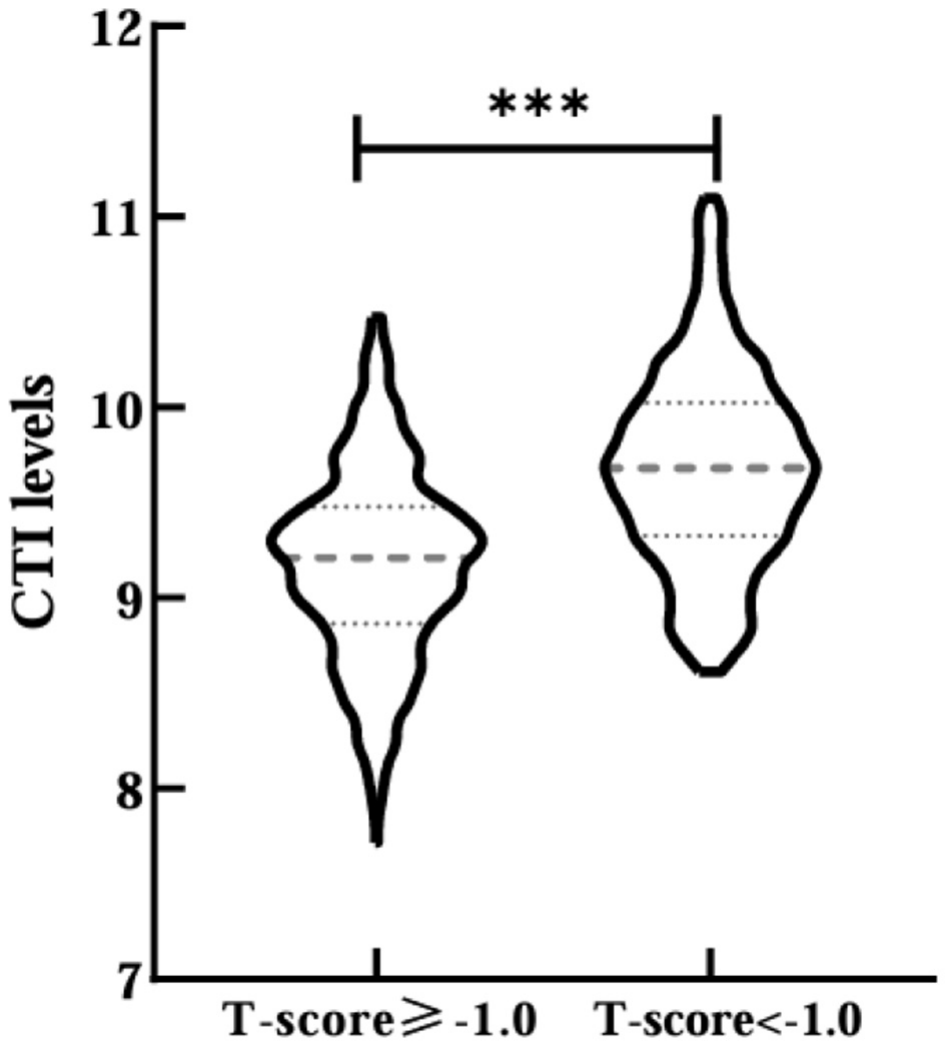
Comparisons of CTI levels between T-score ≥-1.0 and T-score <-1.0 groups. ***Denotes significance at a *p*-value of <0.001.

A total of 847 T2DM patients were included in this study after the exclusion criteria were applied, consisting of 536 male and 311 female. Among these patients, 47.58% (403/847) were diagnosed with osteopenia/OP, which included 189 male and 214 female. Subjects were stratified based on T-score status (T-score ≥-1.0 and T-score < -1.0 groups), with the baseline demographic features and laboratory indices detailed in Table 1. The results demonstrated that compared with the T-score ≥-1.0 group, the subjects in the T-score < -1.0 group, age, HbA1c, FPG, 2h PG, AKP, TC, TG, LDL-C, eGFR, CRP, OC, CTX, PINP, PTH, and CTI (9.179 ± 0.500 vs. 9.684 ± 0.514, *p* < 0.001; Figure 1) levels all increased. In contrast, the percentages of male, T2DM duration, BMI, SLM, FFM, SMM, WHR, FFMI, SMI, Cr, UA, HGB, 25 (OH) D, IGF-1, femur neck BMD, left hip BMD, and lumbar spine BMD levels were significantly lower in the T-score < -1.0 group (*p* < 0.05). And there was a difference in use of insulin and its analog use, GLP-1 receptor agonists, and SGLT-2 inhibitors (*p* < 0.05). No significant differences in other parameters were observed between two groups (*p* > 0.05).

**Table 1:** Characteristics of patients grouped by T-score.

**Variable**	**All (*n* = 847)**	**T-score ≥-1.0 (*n* = 444)**	**T-score < -1.0 (*n* = 403)**	**χ^2^/*t*/*Z***	***p*-Value**
Age (year)	63.19 (58, 68)	62.34 (57, 67)	64.14 (59.70)	−3.892	< 0.001
Male (*n*, %)	536, 63.3%	347, 78.2%	189, 46.9%	88.81	< 0.001
Duration (year)	11.73 (5, 20)	12.29 (6, 20)	11.11 (5, 19)	−2.384	0.017
SBP (mmHg)	133.62 ± 18.13	133.54 ± 17.70	133.71 ± 18.61	0.537	0.895
DBP (mmHg)	80.35 ± 11.05	80.9 ± 11.03	79.74 ± 11.06	0.098	0.128
BMI (kg/m^2^)	24.49 (22.58, 26.25)	24.99 (23.19, 26.78)	23.96 (22.07, 25.66)	−5.316	< 0.001
BFM (kg)	18.44 (14.7, 21.6)	18.81 (15, 21.93)	18.01 (14.35, 21.3)	−1.455	0.146
SLM (kg)	45.35 (38.9, 51)	48.10 (43.2, 52.7)	42.23 (36.65, 47.7)	−10.707	< 0.001
FFM (kg)	48.11 (41.4, 54)	51.00 (51.6, 55.8)	44.83 (39, 50.55)	−10.675	< 0.001
SMM (kg)	26.39 (22.3, 30)	28.16 (25.18, 31)	24.39 (20.95, 27.95)	−10.795	< 0.001
WHR	0.91 (0.88, 0.95)	0.92 (0.88, 0.95)	0.90 (0.87, 0.94)	−3.227	0.001
VFA (cm^2^)	87.85 ± 30.52	88.99 ± 30.52	86.62 ± 30.51	0.116	0.302
FFMI (kg/m^2^)	17.45 (16.2, 18.7)	18.02 (16.9, 19)	16.83 (15.7, 17.9)	−9.039	< 0.001
SMI (kg/m^2^)	7.07 (6.4, 7.7)	7.39 (6.9, 7.9)	6.70 (6.1, 7.4)	−10.6	< 0.001
HbA1c (%)	8.47 (7, 9.55)	8.14 (6.8, 9.3)	8.83 (7.2, 10.1)	−4.912	< 0.001
FPG (mg/dl)	133.85 (106.02, 154.44)	123.52 (101.34, 139.59)	145.24 (115.2, 167.4)	−8.136	< 0.001
2h PG (mg/dl)	263.12 (206.28, 312.3)	251.65 (197.28, 295.7)	275.76 (222.48, 325.08)	−5.212	< 0.001
FINS (μU/ml)	9.63 (3.26, 9.19)	8.84 (3.18, 9.19)	10.54 (3.31, 9.27)	−1.14	0.254
FCP (pmol/L)	584.79 (362, 732)	563.82 (345.5, 718.5)	607.8 (373.75, 757)	−1.236	0.217
ALT (U/L)	22.95 ± 13.64	23.74 ± 13.68	22.08 ± 13.57	1.239	0.078
AST (U/L)	22.45 ± 8.59	22.67 ± 8.39	22.21 ± 8.81	0.005	0.437
γ-GT (U/L)	27.89 (16, 32.4)	26.83 (16.1, 32.4)	29.07 (15.6, 32.78)	−0.064	0.949
AKP (U/L)	76.94 ± 21.56	72.90 ± 20.08	81.41 ± 22.28	5.223	< 0.001
BUN (mmol/L)	6.23 (5, 7.1)	6.25 (5.1, 7.18)	6.21 (5, 6.9)	−0.806	0.42
Cr (umol/L)	68.60 ± 21.97	70.94 ± 18.60	66.03 ± 24.94	10.817	0.001
UA (umol/L)	321 ± 79.12	328.88 ± 75.19	312.33 ± 82.46	0.855	0.002
TC (mg/dl)	170.92 (139.6, 200.31)	163.31 (134.18, 190.26)	179.31 (146.17, 209.59)	−5.129	< 0.001
TG (mg/dl)	134.11 (83.26, 161.2)	127.34 (79.71, 155.66)	141.58 (90.34, 170.94)	−2.731	0.006
HDL-C (mmol/L)	1.10 ± 0.23	1.09 ± 0.24	1.11 ± 0.22	0.414	0.155
LDL-C (mmol/L)	2.60 (2.02, 3.13)	2.48 (1.92, 2.99)	2.75 (2.12, 3.28)	−4.898	< 0.001
Ca (mmol/L)	2.31 ± 0.10	2.31 ± 0.10	2.31 ± 0.10	0.644	0.446
P (mmol/L)	1.14 ± 0.18	1.14 ± 0.18	1.14 ± 0.18	0.158	0.869
eGFR (ml/min/ 1.73 m^2^)	106.2 ± 30.64	104.32 ± 26.68	108.27 ± 34.40	21.784	0.064
CRP (mg/L)	4.39 (2.4, 5.4)	3.71 (2, 4.9)	5.13 (2.7, 6.1)	−6.161	< 0.001
HGB (g/L)	139.31 (132, 151)	141.94 (134, 153)	136.44 (129, 148)	−5.375	< 0.001
CTI	9.419 ± 0.565	9.179 ± 0.500	9.684 ± 0.514	0.657	< 0.001
OC (ng/ml)	13.5 (10.33, 16.27)	13.00 (9.94, 15.58)	14.05 (10.97, 17.01)	−3.997	< 0.001
CTX (ng/ml)	0.39 (0.26, 0.49)	0.35 (0.24, 0.43)	0.44 (0.3, 0.54)	−7.749	< 0.001
PINP (ng/ml)	41.00 (30.11, 49.04)	39.85 (28.86, 46.76)	42.26 (31.73, 51,45)	−3.674	< 0.001
25 (OH) D (ng/ml)	21.82 (16.67, 25.95)	22.89 (17.77, 27.63)	20.64 (16.01, 25)	−4.299	< 0.001
IGF-1 (ng/ml)	118.31 ± 34.46	124.07 ± 32.10	112.05 ± 35.85	0.867	< 0.001
PTH (pmol/L)	5.23 (3.77, 6.19)	5.01 (3.80, 5.86)	5.47 (3.75, 6.56)	−2.259	0.024
Femur neck BMD (g/cm^2^)	0.881 ± 0.135	0.974 ± 0.099	0.778 ± 0.086	6.373	< 0.001
Left hip BMD (g/cm^2^)	0.968 ± 0.143	1.062 ± 0.106	0.864 ± 0.100	0.946	< 0.001
Lumbar spine BMD (g/cm^2^)	1.120 ± 0.182	1.234 ± 0.145	0.995 ± 0.128	1.618	< 0.001
**Anti-diabetic treatment**					
Insulin and its analogs (*n*, %)	374, 44.2%	180, 40.5%	194, 48.1%	4.946	0.026
Biguanides (*n*, %)	660, 78.0%	355, 80.1%	305, 75.7%	2.44	0.118
GLP-1 receptor agonists (*n*, %)	162, 19.1%	104, 23.4%	58, 14.4%	11.14	< 0.001
DPP-IV inhibitors (*n*, %)	336, 39.7%	164, 36.9%	172, 42.7%	2.911	0.088
SGLT-2 inhibitors (*n*, %)	325, 38.4%	190, 42.8%	135, 33.5%	7.717	0.005
Insulin secretagogues (*n*, %)	27, 3.2%	13, 2.9%	14, 3.5%	0.204	0.651
α-glucosidase inhibitors (*n*, %)	202, 23.8%	94, 21.2%	108, 26.8%	3.684	0.055

SBP, systolic blood pressure; DBP, diastolic blood pressure; BMI, body mass index; BFM, body fat mass; SLM, soft lean mass; FFM, fat-free mass; WHR, waist-to-hip ratio; VFA, visceral fat area; ASM, appendicular skeletal muscle mass; FFMI, fat-free mass index; SMI, subsequent appendicular skeletal muscle index (SMI, kg/m^2^); HbA1c, glycated hemoglobin; FPG, fasting plasma glucose; 2h PG, 2-h post-load plasma glucose; FINS, fasting insulin; FCP, fasting C-peptide; ALT, alanine aminotransferase; AST, aspartate aminotransferase; γ-GT, γ-glutamyl transferase; AKP, alkaline phosphatase; BUN, blood urea nitrogen; Cr, creatinine; UA, uric acid; TC, triglyceride; TG, triglyceride; HDL-C, high-density lipoprotein cholesterol; LDL-C, low-density lipoprotein cholesterol; Ca, serum calcium; P, serum phosphorus; eGFR, estimated glomerular filtration rate; CRP, C-reactive protein; HGB, hemoglobin; CTI, C-reactive protein-triglyceride glucose index; OC, osteocalcin; CTX, Beta-isomer of the carboxy-terminal cross-linked telopeptide of type I collagen; PINP, procollagen type I N-terminal propeptide; 25 (OH) D, 25-hydroxyvitamin D; IGF-1, insulin-like growth factor-1; PTH, parathyroid hormone; BMD, bone mineral density; GLP-1, glucagon-like peptide-1; DPP-IV, dipeptidyl peptidase-4; SGLT-2, sodium-glucose co-transporter-2.

**Figure 2:**
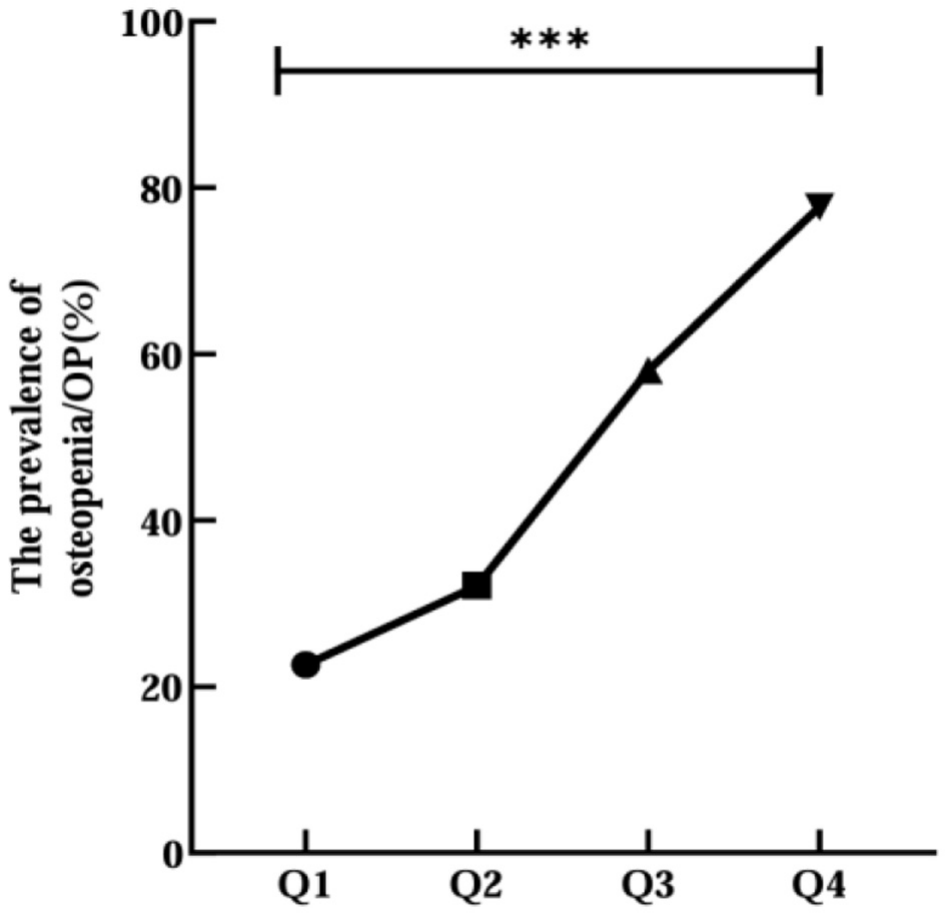
Relationship between CTI quartile and the prevalence of OP or osteopenia. ***Denotes significance at a *p*-value of <0.001.

Then, all participants were divided into four groups (Q1 < 9.036, Q2 9.036–9.397, Q3 9.398–9.779, Q4 > 9.779) by CTI quartiles. The prevalence of osteopenia/OP increased progressively with elevating CTI levels (Q1 48, 22.6%; Q2 68, 32.1%; Q3 123, 58.0%; Q4 164, 77.7%; *p* < 0.001; Figure 2). There were significant differences in age, the percentages of male, SLM, FFM, SMM, SMI, HbA1c, FPG, 2h PG, AKP, Cr, TC, TG, LDL-C, CRP, HGB, OC, 25 (OH) D, IGF-1, PTH, FN BMD, LH BMD, LS BMD, and use of insulin and its analogs among three groups (*p* < 0.05; Table 2). There was no notable variation in other characters between normal and abnormal BMD patients with T2DM (*p* > 0.05; Table 2).

**Table 2:** Characteristics of patients grouped by CTI levels.

**Variable**	**Q1 (*n* = 212)**	**Q2 (*n* = 212)**	**Q3 (*n* = 211)**	**Q4 (*n* = 212)**	**χ^2^/F/H**	***p*-Value**
T-score < – 1.0 (*n*, %)	48, 22.6%	68, 32.1%	123, 58.0%	164, 77.7%	159.436	< 0.001
CTI	8.713 ± 0.271	9.236 ± 0.101	9.586 ± 0.114	10.146 ± 0.302	793.125	< 0.001
Age (year)	62.01 (58, 66)	62.77 (57, 68)	63.33 (57, 69)	64.67 (60, 70)	16.112	0.001
Male (*n*, %)	154, 72.6%	150, 70.8%	118, 55.7%	114, 54.0%	26.163	< 0.001
Duration (year)	12.12 (5, 20)	11.52 (5, 19)	11.75 (5, 20)	11.52 (5, 20)	0.853	0.837
SBP (mmHg)	131.42 ± 17.25	132.95 ± 19.48	135.2 ± 17.51	134.91 ± 18.06	7.461	0.059
DBP (mmHg)	81.06 ± 10.42	80.12 ± 11.61	80.56 ± 11.87	79.64 ± 10.24	1.796	0.616
BMI (kg/m^2^)	24.34 (22.25, 26.12)	24.48 (22.58, 26.37)	24.68 (22.87, 26.2)	24.46 (22.58, 26.33)	1.028	0.751
BFM (kg)	17.96 (14.4, 20.9)	18.26 (14.73, 21.28)	18.86 (15.1, 22.5)	18.67 (15.2, 22.05)	3.571	0.312
SLM (kg)	47.06 (41.1, 52.4)	46.05 (40.55, 51.78)	44.88 (38.8, 50.7)	43.33 (37.5, 48.85)	26.233	< 0.001
FFM (kg)	49.91 (43.7, 55.6)	48.84 (43.08, 54.78)	47.62 (41.2, 53.6)	45.99 (39.85, 51.8)	25.945	< 0.001
SMM (kg)	27.5 (24, 30.9)	26.88 (23.2, 30.4)	26.07 (22, 29.7)	25.07 (21.5, 28.8)	27.737	< 0.001
WHR	0.91 (0.88, 0.94)	0.91 (0.88, 0.95)	0.91 (0.88, 0.95)	0.91 (0.87, 0.95)	2.297	0.513
VFA (cm^2^)	85.51 ± 29.20	86.22 ± 28.38	90.21 ± 34.12	89.57 ± 30.21	2.737	0.434
FFMI	17.7 (16.5, 19)	17.5 (16.2, 18.85)	17.34 (16.1, 18.6)	17.23 (16.1, 18.3)	8.277	0.041
SMI	7.23 (6.6, 7.8)	7.14 (6.5, 7.8)	7.01 (6.3, 7.7)	6.88 (6.3, 7.5)	20.399	< 0.001
HbA1c (%)	7.85 (6.6, 8.7)	8.35 (7, 9.4)	8.45 (7, 9.4)	9.19 (7.4, 10.63)	52.934	< 0.001
FPG (mg/dl)	111.59 (95.09, 123.44)	127.80 (103.73, 147.74)	135.10 (108.05, 153.9)	161.05 (123.66, 184.5)	164.841	< 0.001
2h PG (mg/dl)	238.14 (183.42, 282.29)	254.72 (204.03, 304.61)	261.01 (213.84, 309.69)	298.73 (230.94, 341.46)	61.939	< 0.001
FINS (μU/ml)	7.50 (3.01, 8.77)	12.21 (3.28, 8.85)	8.78 (3.32, 10.1)	9.96 (3.37, 9.14)	4.233	0.237
FCP (pmol/L)	551.24 (337, 691.5)	578.36 (373.25, 711)	593.34 (346.5, 806.25)	616.04 (380.25, 757.75)	3.55	0.314
ALT (U/L)	23.16 ± 14.25	23.50 ± 13.53	22.93 ± 12.66	22.20 ± 14.13	3.048	0.384
AST (U/L)	22.25 ± 7.98	22.89 ± 8.78	22.50 ± 8.24	22.16 ± 9.35	2.491	0.477
γ-GT (U/L)	25.50 (16, 29.3)	29.38 (15.75,34.85)	26.69 (16.08,29.53)	30.10 (16.05, 33.5)	2.862	0.413
AKP (U/L)	70.44 ± 19.17	76.81 ± 19.06	76.49 ± 21.31	84.08 ± 24.26	41.982	< 0.001
BUN (mmol/L)	6.25 (5, 7.1)	6.24 (5.2, 6.98)	6.31 (5, 7.38)	6.13 (4.8, 7)	2.94	0.401
Cr (umol/L)	69.36 ± 17.95	69.23 ± 18.96	69.27 ± 24.53	66.55 ± 25.48	10.538	0.015
UA (umol/L)	329.66 ± 75.76	322.67 ± 75.27	313.69 ± 76.66	318 ± 87.81	7.095	0.069
TC (mg/dl)	161.99 (132.45, 185.81)	168.12 (135.25, 199.83)	172.72 (141.24, 202.44)	180.92 (150.81, 180.00)	22.436	< 0.001
TG (mg/dl)	118.05 (74.62, 143.62)	129.55 (83.26, 161.20)	142.79 (88.79, 168.06)	146.13 (91.23, 179.80)	23.428	< 0.001
HDL-C (mmol/L)	1.11 ± 0.24	1.08 ± 0.23	1.10 ± 0.22	1.12 ± 0.23	2.938	0.401
LDL-C (mmol/L)	2.43 (1.85, 2.91)	2.55 (1.95, 3.10)	2.65 (2.02, 3.19)	2.79 (2.2, 3.31)	24.191	< 0.001
Ca (mmol/L)	2.30 ± 0.09	2.31 ± 0.09	2.31 ± 0.10	2.31 ± 0.11	1.66	0.646
P (mmol/L)	1.14 ± 0.18	1.14 ± 0.18	1.14 ± 0.17	1.12 ± 0.18	1.849	0.604
eGFR (ml/min/ 1.73 m^2^)	105.73 ± 27.35	104.91 ± 25.73	104.73 ± 34.08	109.45 ± 34.4	3.935	0.269
CRP (mg/L)	2.86 (1.5, 3.98)	4.06 (2.5, 5.3)	4.86 (3, 6)	5.76 (3, 7.1)	115.255	< 0.001
HGB (g/L)	139.56 (132, 151)	142.1 (134, 154.25)	136.95 (129, 149)	138.65 (131, 149)	16.731	< 0.001
OC (ng/ml)	13.16 (10.34, 15.53)	13.53 (10.32, 16.91)	13.37 (10.57, 15.58)	13.95 (10.21, 17.01)	2.22	0.528
CTX (ng/ml)	0.36 (0.24, 0.46)	0.40 (0.26, 0.48)	0.39 (0.29, 0.49)	0.41 (0.26, 0.53)	11.191	0.011
PINP (ng/ml)	39.48 (28.86, 46.13)	40.96 (29.1, 49.43)	42.06 (31.75, 49.47)	41.53 (29.64, 50.61)	4.629	0.201
25 (OH) D (ng/ml)	22.84 (17.86, 27.88)	22.42 (17.54, 27.24)	21.02 (15.83, 24.87)	21.01 (16.16, 25.27)	12.659	0.005
IGF-1 (ng/ml)	123.01 ± 33.67	120.22 ± 33.62	117.21 ± 33.77	112.84 ± 36.09	10.051	0.018
PTH (pmol/L)	5.16 (3.89, 5.83)	5.29 (3.85, 6.43)	5.14 (3.72, 5.98)	5.34 (3.66, 6.22)	1.818	0.611
Femur neck BMD (g/cm^2^)	0.925 ± 0.118	0.905 ± 0.138	0.866 ± 0.138	0.827 ± 0.126	76.354	< 0.001
Left hip BMD (g/cm^2^)	1.009 ± 0.123	0.989 ± 0.148	0.956 ± 0.148	0.916 ± 0.134	63.099	< 0.001
Lumbar spine BMD (g/cm^2^)	1.174 ± 0.163	1.155 ± 0.181	1.099 ± 0.180	1.050 ± 0.176	70.149	< 0.001
**Anti-diabetic treatment**						
Insulin and its analogs (*n*, %)	79, 37.3%	78, 36.8%	101, 47.6%	116, 55.0%	19.808	< 0.001
Biguanides (*n*, %)	165, 77.8%	169, 79.7%	156, 73.9%	170, 80.6%	3.214	0.36
GLP-1 receptor agonists (*n*, %)	41, 19.3%	38, 17.9%	41, 19.3%	42, 19.9%	0.293	0.961
DPP-IV inhibitors (*n*, %)	81, 38.2%	85, 40.1%	88, 41,5%	82, 38.9%	0.563	0.905
SGLT-2 inhibitors (*n*, %)	79, 37.3%	80, 37.7%	84, 39.6%	82, 38.9%	0.308	0.959
Insulin secretagogues (*n*, %)	6, 2.8%	3.3, 3.3%	8, 3.8%	6, 2.8%	0.414	0.937
α-glucosidase inhibitors (*n*, %)	43, 20.3%	49, 23.1%	53, 25.0%	57, 27.0%	2.866	0.413

**Figure 3:**
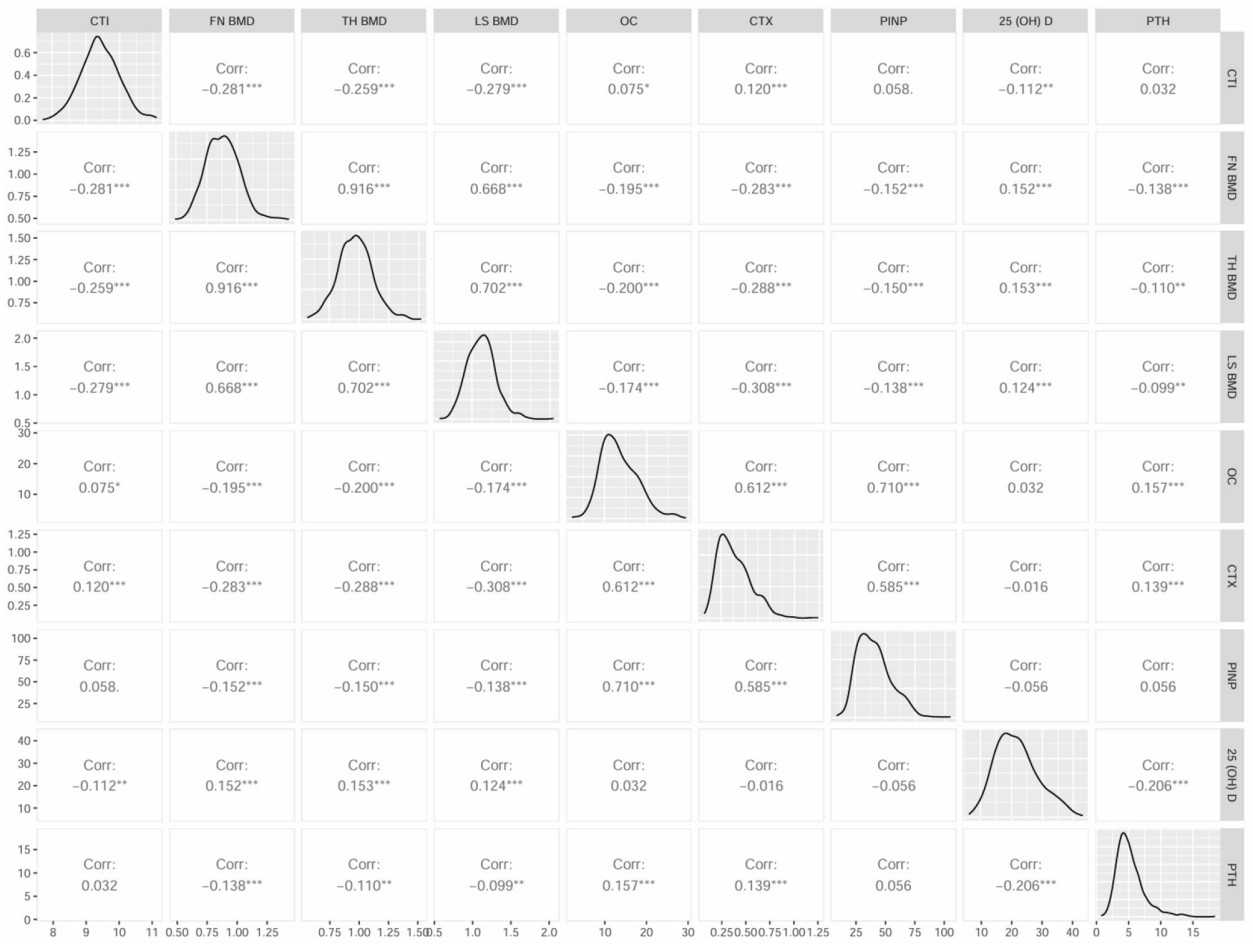
Matrix scatter diagram by Spearman's test for CTI and bone turnover markers (BTMs), BMD parameters.

Further, Spearman's correlation analysis was adopted to explore the connection of CTI with bone turnover markers (BTMs) and BMDs. The results were shown in Figure 3, which revealed positive correlation between CTI and OC (*r* = 0.075), CTX (*r* = 0.120), whereas significantly negative correlation with FN BMD (*r* = −0.281), LH BMD (*r* = −0.259), LS BMD (*r* = −0.279), and 25 (OH) D (*r* = −0.112) in all subjects (*p* < 0.05). Moreover, there was no correlation between CTI and PINP (*r* = 0.058) and PTH (*r* = 0.032; *p* > 0.05). The correlation of CTI and BMDs in male and female was also performed (Supplementary Table 2).

In order to assess the overall osteopenia/OP risk at the CTI levels and quartiles, logistic analyses were conducted (Table 3). In regression analysis of CTI as a continuous variable, it was shown that CTI levels were significantly associated with the risk of osteopenia/OP both in unadjusted and adjusted models for all subjects in this study (Table 3). With the CTI as a stratified indicator, in unadjusted model (model 1) compared with Q1, Q2 (Q2 vs. Q1, OR, 1.613; 95% CI: 1.047–2.458), Q3 (Q3 vs. Q1, OR, 4.722; 95% CI: 3.097–7.199) and Q4 (Q4 vs. Q1, OR, 11.922; 95% CI: 7.511–18.824) all kept an independent effect on osteopenia/OP presence. After age and gender adjustment, in comparison to Q1 group, patients in Q2, Q3 and Q4 all had a significantly increased risk of DR by 61.5% (OR, 1.615; 95% CI: 1.029–2.536), 323.9% (OR, 4.239; 95% CI: 2.725–6.594) and 1,011% (OR, 11.11; 95% CI: 6.895–17.902), respectively. After further adjustment for T2DM duration, BMI, SLM, FFM, SMM, FFMI, SMI, HbA1c, 2h PG, AKP, UA, TC, LDL-C, HGB, IGF-1, OC, CTX, PINP, 25 (OH)D, PTH, and use of insulin and its analogs, GLP-1 receptor agonists, SGLT-2 inhibitors, compared to the Q1 of CTI, subjects in Q3 and Q4 still had a remarkably increased risk of DR (OR 4.061, 11.086, separately). The relationship of CTI and the overall osteopenia/OP risk in male and female was shown in Supplementary Table 3.

**Table 3:** Logistic regression analysis of CTI of OP or osteopenia.

**Variables**	**Model 1**			**Model 2**			**Model 3**		
	**OR**	**95% CI**	***p*** **-value**	**OR**	**95% CI**	***p*** **-Value**	**OR**	**95% CI**	***p*** **-Value**
CTI	7.42	5.305, 10.378	< 0.001	6.907	4.877, 9.783	< 0.001	7.277	4.602, 11.507	< 0.001
**CTI (quartile)**									
**Q1**									
Q2	1.613	1.047, 2.485	0.03	1.615	1.029, 2.536	0.037	1.175	0.662, 2.088	0.581
Q3	4.722	3.097, 7.199	< 0.001	4.239	2.725, 6.594	< 0.001	4.061	2.305, 7.155	< 0.001
Q4	11.922	7.511, 18.824	< 0.001	11.11	6.895, 17.902	< 0.001	11.086	5.947, 20.666	< 0.001

OR, odds ratio; CI, confidence interval.

Model 1: no covariates were adjusted.

Model 2: adjusted for age and gender.

Model 3: adjusted for age, gender, duration, BMI, SLM, FFM, SMM, FFMI, SMI, HbA1c, 2h PG, AKP, UA, TC, LDL-C, HGB, IGF-1, OC, CTX, PINP, 25 (OH)D, PTH, and treatment of insulin and its analogs, GLP-1 receptor agonists, SGLT-2 inhibitors.

**Figure 4:**
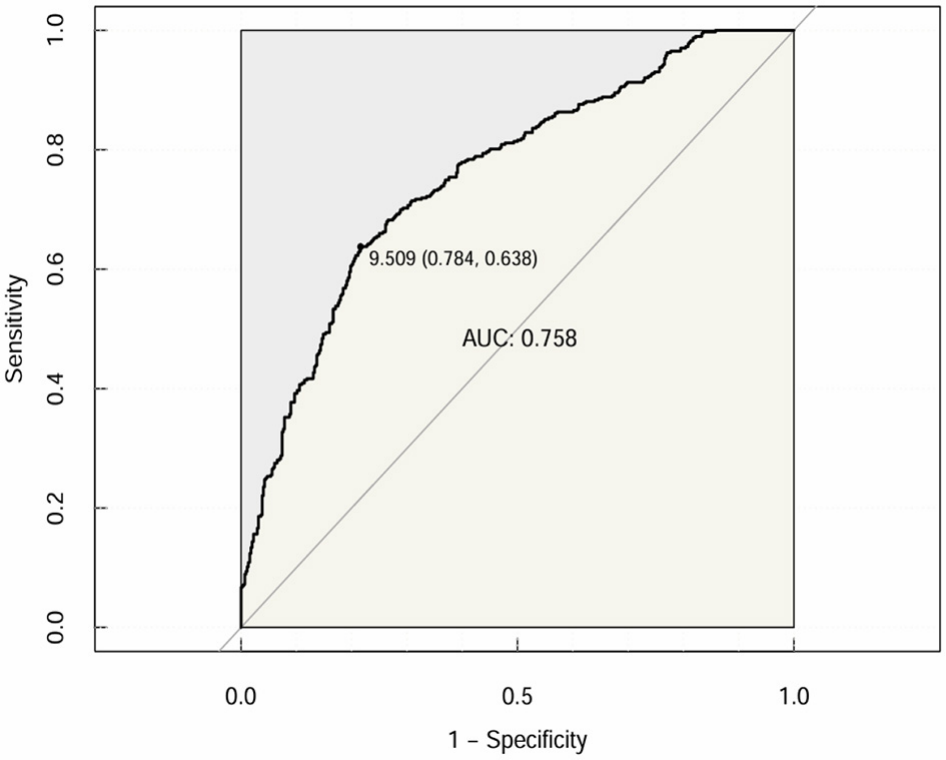
CTI for predicting the risk of OP or osteopenia in T2DM patients.

Furthermore, CTI was subjected to receiver operating characteristic (ROC) curve analysis to assess its predictive value for OP or osteopenia in elderly individuals with T2DM. The area under the curve (AUC) of the CTI was 0.758 (95% CI: 0.7254–0.7897; specificity = 69.3%; sensitivity = 71.2%), with a Youden index of 9.509 (Figure 4).

## Discussion

Our study examined the relationship between C-reactive protein-triglyceride-glucose index (CTI) levels and osteopenia/OP in middle-aged and elderly individuals with T2DM. Overall, the CTI demonstrated a significant positive association with OP/osteopenia risk and remained significantly associated after controlling for a few confounding factors. By ROC curve analysis, this study revealed that the CTI could serve as a novel and moderate predictor for screening osteopenia/osteoporosis in middle-aged and elderly patients with T2DM.

DM is a prevalent chronic disease that poses a substantial threat to global health and carries an enormous social and economic burden (8). Bone loss is a natural consequence of aging, particularly beyond middle age (6). Compared with the normal population, patients with T2DM have a higher risk of bone metabolism abnormality and fracture (10). The elevated risk is attributed to the complex bidirectional relationship between T2DM and bone mass abnormalities. On the one hand, T2DM directly inhibits osteoblast function and interferes with the balance of bone metabolism through oxidative stress, inflammation, and metabolic disorders caused by hyperglycemia, leading to a decrease in bone mass and an increase in the risk of osteoporosis; on the other hand, bone mass abnormalities can further exacerbate insulin resistance and glycemic abnormality, while glycemic abnormality can increase the risk of osteoporosis by affecting insulin resistance and glycemic abnormality, forming a vicious circle (10, 13, 24). This highlights the urgent need for effective risk stratification of this population to improve its management and prognosis, especially in the middle-aged and elderly population.

The TyG index, derived from TG and FPG levels, is a practical and easily applicable tool in clinical settings. Elevated FPG and TG levels are two components of the metabolic syndrome associated with IR and the development of chronic disease. IR is a critical factor in many metabolic disorders, and the TyG index has been shown to reflect underlying IR. Recent studies have found significant associations between the TyG index and T2DM and related complications (15, 16). C-reactive protein (CRP), the first acute phase protein to be used in the clinical laboratory, is a greatly sensitive systemic marker of inflammation and tissue injury. As an acute phase protein, it is synthesized by the liver during the secretion of a variety of inflammatory cytokines, including interleukin 6 (IL-6), IL-1, and tumor necrosis factor (TNF). The growing researches suggested that elevated levels of CRP are strongly associated with the risk of metabolic disease, cardiovascular disease, and certain cancers (25, 26). In our research, we also found the increasing trend in FPG, TG and CRP levels in T2DM patients in T-score < -1.0 group.

The complexity of the endocrine system increases significantly with age, especially in the presence of multiple chronic diseases. While single markers like CRP or the TyG index offer valuable insights into isolated aspects of inflammation or IR, they often fall short of capturing the complex, intertwined nature of metabolic pathologies. The CTI integrates both dimensions—IR (a key metabolic compartment) and systemic inflammation—into a single metric. This integration provides a more holistic view of the pathophysiological milieu that drives bone loss in diabetes. It reflects the synergistic damage caused by both metabolic and inflammatory pathways, thereby offering a more comprehensive tool for risk stratification than any single marker could achieve alone. Therefore, a composite assessment system incorporating both IR and inflammation indicators can more comprehensively reflect the integrated dynamic changes in the body's metabolism and inflammation, providing a more accurate basis for disease diagnosis and management.

CTI is a recently introduced index designed to assess inflammation (via CRP) and insulin resistance (via TyG), both of which are risk factors for DM and osteopenia/OP, and it is a direct, rapid indicator at no additional cost beyond standard laboratory tests. According to the present study, there was a progressively increasing prevalence of osteopenia/osteoporosis with higher quartile groups (*p* for trend < 0.05). Moreover, a strongly correlation was shown between CTI and the likelihood of osteopenia/OP, when CTI Q1 was used as the reference, the OR for osteopenia/OP risk was significant risen for CTI Q3 and Q4 after adjusted for covariates. Osteopenia/OP is characterized by reduced BMD and deterioration of bone microarchitecture, and T2DM is associated with impaired microarchitecture and bone mass. Several investigations have discovered that IR status is tightly regulated with poor bone turnover and imbalanced bone resorption and formation (27–29). Previous study found that CRP may have a direct role on osteoclast and osteoblast differentiation via TLR signaling pathways (27). A study reported a notable negative correlation between CRP and total bone trabecular score (TBS). These findings implied that IR and inflammation may be potentially linked in the pathogenesis affecting osteopenia/OP, providing further insights into the common mechanisms between the diseases.

The findings of our study underscored the importance of CTI as a comprehensive indicator for predicting osteopenia/OP risk in T2DM patients. The significant association between CTI and osteopenia/OP risk, even after adjusting for multiple confounding factors, suggests that CTI can effectively integrate the effects of IR and inflammation on bone health. This composite index may offer a more holistic approach to risk assessment compared to single biomarkers, which often fail to capture the multifactorial nature of metabolic and inflammatory processes in chronic diseases.

In addition, although our study focused on the metabolic and inflammatory pathways captured by CTI, the observed higher prevalence of osteopenia/OP in female is consistent with the well-established role of menopause and estrogen deficiency in bone biology. The rapid decline in estrogen levels during menopause accelerates bone resorption by disrupting the balance between osteoclast and osteoblast activity, leading to a period of accelerated bone loss (2, 30–32). This fundamental biological difference explains the higher baseline risk in women. The novel and critical insight from our study, however, is that the association between CTI and osteopenia/OP risk was consistent and independent of gender (Supplementary Table 3). This suggests that while the absolute risk of OP is higher in female due to hormonal factors, the metabolic and inflammatory risk quantified by CTI operates through a parallel pathway that is equally relevant to both sexes. Therefore, CTI does not replace the established understanding of hormonal bone loss but rather complements it by providing a sex-agnostic tool to identify individuals at risk due to dysmetabolism and inflammation. This is particularly valuable for risk stratification in male populations and in postmenopausal women where both hormonal and metabolic pathways can contribute synergistically to bone loss.

Furthermore, the findings of our study carry significant implications for clinical management. The CTI, derived from routine laboratory data, which could prompt clinicians to initiate earlier screening. Furthermore, CTI holds potential to supplement established fracture risk assessment tools like FRAX. While FRAX incorporates clinical risk factors and BMD, it does not specifically account for the interplay of diabetes-specific factors like insulin resistance and chronic inflammation. CTI could therefore serve as a valuable, disease-specific enhancer to FRAX, particularly in refining risk prediction in the T2DM population. Nevertheless, this study had several limitations, the first of which was its cross-sectional design, making it difficult to determine causation between CTI and osteopenia/OP in T2DM patients. Second, the sample size of this study was relatively small and single center. Third, the gender distribution in our study was imbalanced (~63% male), which may limit the generalizability of our results, especially to postmenopausal female populations where osteoporosis is most prevalent. However, the consistent performance of CTI across both sexes, as demonstrated in our stratified analyses, strengthens confidence in its utility as a general metabolic indicator (Supplementary Table 1). Future studies with a larger, balanced sample size and a specific focus on high-risk populations, such as postmenopausal women, are warranted to further validate and refine its application. Finally, although we adjusted for some of the confounders, there may still be unmeasured confounding factors may affect our research results, such as dietary habits, sunlight exposure time, and amount of exercise. Therefore, future cohorts with larger sample sizes are needed to further analyze this association. What is more, basic research is needed to explore the mechanism of the interaction of IR-inflammation on osteoblasts/osteoclasts and bone metabolism.

## Conclusion

CTI is independently associated with abnormal BMD in patients with T2DM. Given its moderate predictive accuracy, CTI may be regarded a novel, supportive biomarker to complement existing tools for identifying individuals at high risk of osteopenia and osteoporosis. Attention should be paid to the role of IR and inflammation in the relationship between CTI and metabolic disorders. Meanwhile, our current study revealing that timely monitoring and regular evaluation of CTI in clinical practice could be clinically valuable for the early prevention of bone metabolic complications in elderly T2DM patients. Future prospective studies, particularly those designed to develop and validate sex-stratified risk models incorporating CTI, are warranted to confirm its generalizability and translational utility.

## Data Availability

The original contributions presented in the study are included in the article/Supplementary Material, further inquiries can be directed to the corresponding author.
