# Supplementary material for: Association between C-reactive protein-triglyceride glucose index and abnormal BMD in middle-aged and elderly patients with type 2 diabetes mellitus: a cross-sectional study
Source: Front Med (Lausanne). 2025 Oct 16;12:1615596. doi: 10.3389/fmed.2025.1615596 (PMC12571813; doi:10.3389/fmed.2025.1615596)
Supplement: Supplementary file 1 [file Data_Sheet_1.PDF]

Table 1 Characteristics of patients grouped by T-score in male and female.

Variables	Male(n=536)			Female(n=311)		
	T-score≥-1.0 (n=347)	T-score<-1.0 (n=189)	p-value	T-score≥-1.0 (n=97)	T-score<-1.0 (n=214)	p-value
FPG (mg/dl)	124.47±34.44	138.44±38.73	<0.001	120.12±33.93	151.22±46.14	<0.001
TG (mg/dl)	124.85±65.72	135.27±80.63	0.333	136.22±72.97	147.12±74.57	0.224
CRP (mg/l)	3.67±2.35	4.81±3.34	<0.001	3.88±2.07	5.41±4.10	0.001
CTI	9.18±0.51	9.65±0.53	<0.001	9.18±0.468	9.72±0.502	<0.001
OC (ng/ml)	12.42±3.80	13.24±4.19	0.01	15.00±4.07	14.77±4.66	0.593
CTX (ng/ml)	0.32±0.14	0.42±0.18	<0.001	0.42±0.14	0.45±0.16	0.286
PINP (ng/ml)	37.14±13.07	38.87±12.38	0.02	49.39±15.61	45.22±14.31	0.12
25 (OH) D (ng/ml)	23.52±7.05	21.50±6.74	0.004	20.65±6.30	19.88±6.51	0.473
IGF-1 (ng/ml)	126.15±31.99	119.75±36.87	0.04	116.54±31.52	105.30±33.60	0.006
PTH (pmol/L)	5.06±2.01	5.31±2.30	0.293	4.84±1.62	5.62±2.47	0.069
FN BMD (g/cm^2)	0.984±0.101	0.795±0.069	<0.001	0.940±0.085	0.762±0.097	<0.001
LH BMD (g/cm^2)	1.070±0.105	0.887±0.089	<0.001	1.031±0.106	0.842±0.104	<0.001
LS BMD (g/cm^2)	1.248±0.151	1.177±0.171	<0.001	1.176±0.016	0.948±0.113	<0.001

Table 2 Correlation analysis of CTI and BMDs in male and female.

Variables	male		female	
	r	p	r	p
FN BMD (g/cm^2)	-0.254	<0.001	-0.207	<0.001
LH BMD (g/cm^2)	-0.23	<0.001	-0.185	0.001

LS BMD (g/cm^2)	-0.205	<0.001	-0.286	<0.001

Table 3 Logistic regression analysis of CTI of OP or osteopenia in male and female.

A. CTI as continuous variable.

CTI	Model 1		Model 2	
	OR (95%CI)	p-value	OR (95%CI)	p-value
male	5.882(3.911,8.847)	<0.001	7.387(4.207,12.973)	<0.001
Female	10.622(5.523,20.427)	<0.001	9.983(4.306,18.473)	<0.001

B. CTI as categorized quartiles.

CTI (quartile)		Model 1		Model 2	
		OR (95%CI)	p-value	OR (95%CI)	p-value
male	Q1				
	Q2	1.443(0.817,2.549)	0.207	1.016(0.487,2.121)	0.866
	Q3	4.447(2.552,7.748)	<0.001	4.499(2.205,9.179)	<0.001
	Q4	8.765(4.961,15.489)	<0.001	10.505(4.930,18.388)	<0.001
female	Q1				
	Q2	1.987(0.958,4.119)	0.055	1.578(0.593,4.201)	0.361
	Q3	4.061(2.030,8.124)	<0.001	3.759(1.408,10.039)	0.008
	Q4	16.818(6.299,26.241)	<0.001	12.946(4.707,20.001)	<0.001

Model 1: no covariates were adjusted;

Model 2: adjusted for age, duration, BMI, SLM, FFM, SMM, FFMI, SMI, HbA1c, 2h PG, AKP, UA, TC, LDL-C, HGB, IGF-1, OC, CTX, PINP, 25(OH)D, PTH, and treatment of insulin and its analogs, GLP-1 receptor agonists, SGLT-2 inhibitors.
